# HIV-1 Viral Load Assays for Resource-Limited Settings: Authors' Reply

**DOI:** 10.1371/journal.pmed.0030550

**Published:** 2006-12-26

**Authors:** Susan A Fiscus

We thank Drs. Preiser, Drexler, and Drosten for their letter [1] and share their concerns regarding HIV-1 viral load assays in resource-limited settings. The real-time RT-PCR (reverse-transcriptase polymerase chain reaction) assay they have developed had not yet been published when our review was submitted or revised. As noted in Table 1 of our review, many of the current viral load assays require additional evaluation with different subtypes and others may underestimate non-B subtypes. In addition, under the heading “What is needed for implementation of simplified viral load testing?” we state “First, each country must determine if the assay will quantify subtypes common in the region and is appropriate for the technical staff and laboratory equipment available.”

In-house assays require production, optimization, validation, and quality assurance of all reagents, which is challenging in any circumstances, and would be extremely difficult in laboratories in most resource-limited settings. Real-time PCR instruments are expensive and maintenance in many places would be a problem. What is really needed is a technologically much simpler, much less expensive, perhaps semi-quantitative assay for measuring viral load.
